# Carbon dioxide capture and conversion by an acid-base resistant metal-organic framework

**DOI:** 10.1038/s41467-017-01166-3

**Published:** 2017-11-01

**Authors:** Linfeng Liang, Caiping Liu, Feilong Jiang, Qihui Chen, Linjie Zhang, Hui Xue, Hai-Long Jiang, Jinjie Qian, Daqiang Yuan, Maochun Hong

**Affiliations:** 1 State Key Laboratory of Structure Chemistry, Fujian Institute of Research on the Structure of Matter, Chinese Academy of Sciences, Fuzhou, Fujian, 350002 China; 2 Department of Chemistry, University of Science and Technology of China, Hefei, Anhui 230026 China; 3 Hefei National Laboratory for Physical Sciences at the Microscale, Hefei, Anhui 230026 China; 4 College of Chemistry and Materials Engineering, Wenzhou University, Wenzhou, 325035 China

## Abstract

Considering the rapid increase of CO_2_ emission, especially from power plants, there is a constant need for materials which can effectively eliminate post-combustion CO_2_ (the main component: CO_2_/N_2_ = 15/85). Here, we show the design and synthesis of a Cu(II) metal-organic framework (**FJI-H14**) with a high density of active sites, which displays unusual acid and base stability and high volumetric uptake (171 cm^3^ cm^−3^) of CO_2_ under ambient conditions (298 K, 1 atm), making it a potential adsorbing agent for post-combustion CO_2_. Moreover, CO_2_ from simulated post-combustion flue gas can be smoothly converted into corresponding cyclic carbonates by the **FJI-H14** catalyst. Such high CO_2_ adsorption capacity and moderate catalytic activity may result from the synergistic effect of multiple active sites.

## Abstract (web summary)

Increasing CO_2_ emissions pose serious environmental issues. Here, the authors report the synthesis of a robust metal-organic framework which displays high volumetric uptake of post-combustion CO_2_ under ambient conditions and can catalyze CO_2_ fixation into cyclic carbonates.

## Introduction

Due to the dependence on fossil fuels to meet the world’s growing energy demand, the concentration of CO_2_ in the atmosphere has increased from 310 p.p.m. to over 380 p.p.m. during the last half century, and it continues to rise^1–3^. Some 60% of the total CO_2_ emission worldwide is attributable to electricity generation^4^. The installation of effective CO_2_ capture systems that can selectively remove the CO_2_ component of the exhaust gas emitted by coal- or gas-fired power plants would extremely reduce the global annual emissions. Current technologies involving aqueous amine absorbents for the capture of CO_2_ from a gas mixture are usually themselves significant consumers of energy and a source of corrosion problems in equipment^5–7^. Thus, exploration of new materials that can selectively and efficiently eliminate combustion-generated CO_2_ is urgent.

Due to their large capacity for the adsorption of gases and their chemical tunability^8–21^, the emerging porous metal-organic frameworks (MOFs) could serve as promising cost-effective and efficient materials for CO_2_ capture and separation, but development of practically useful MOF materials for CO_2_ capture directly from power plants remains a challenge. The flue gas generated from coal-fired power plant, released at a total pressure of approximately 1 atm., contains 15–16% CO_2_, 73–77% N_2_, 5–7% H_2_O, 3–4% O_2_ and a small amount of acid gas^22–24^. An ideal MOF material for CO_2_ capture should exhibit extraordinarily high CO_2_ uptake and selectivity at ambient pressures; furthermore, it should also be resistant to water and acid gas, can be prepared on a large scale and is reusable. Moreover, in terms of practical applications, high volumetric CO_2_ adsorption capacity seems even more important than gravimetric CO_2_ adsorption capacity, since the capture and separation of post-combustion CO_2_ is often carried out in a fixed-bed reactor^5^.

Generally, open metal sites (OMS) and Lewis basic sites (LBS) are favorable for interaction with CO_2_ and various types of OMS and LBS have been introduced into MOFs in an effort to improve their adsorption capacity^25–32^. To adsorb CO_2_ effectively under ambient conditions, one would seek to prepare an MOF with high densities of OMS and LBS, in which the OMS and LBS could synergistically capture CO_2_ molecules in the pores. Such a potential synergy effect from the OMS and LBS can improve the adsorption capacity more effectively than a single OMS or LBS.

In this paper, we describe the design and synthesis of a Cu(II)-MOF, **FJI-H14** with a high density of OMS and LBS, which shows extraordinary high volumetric uptake of CO_2_ at ambient conditions and excellent selectivity for CO_2_ over N_2_. Remarkably, it is highly stable in a water and acid/base environment and can be reused without loss of adsorption capacity; furthermore, it can be easily synthesized in large quantities. Experiments with simulated post-combustion flue gas have shown that **FJI-H14** can smoothly catalyze the chemical transformation of CO_2_ into the corresponding cyclic carbonates.

## Results

### Synthesis and structure analysis

**Fig. 1:**
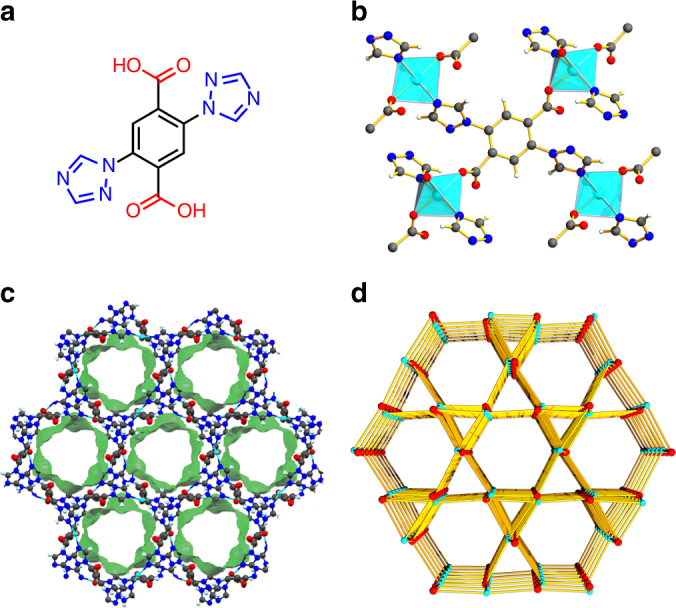
Structural illustration of FJI-H14. **a** The selected ligand H_2_BTTA for the construction of **FJI-H14**. **b** The coordination environment of the Cu(II) ions as four-connected nodes and BTTA also as a four-connected node. **c** The one-dimensional nanoporous channels along the crystallographic *c* direction. **d** The framework of **USF** topology. (Cu atom, cyan; C atom, gray; O atom, red; N atom, blue; H atom, white)

The reaction of 2,5-di(1*H*-1,2,4-triazol-1-yl)terephthalic acid (H_2_BTTA, Fig. 1a) with Cu(NO_3_)_2_ in H_2_O at 120 °C for 3 days affords rod-shaped blue crystals of **FJI-H14** ([Cu(BTTA)H_2_O]_n_·6nH_2_O) in 73% yield. Single-crystal X-ray structure analysis shows that **FJI-H14** crystallizes in trigonal space group *R*-3 (for more details see Supplementary Table 1). The crystallographic asymmetric unit contains one BTTA^2−^ligand, one Cu(II) ion and one coordinate water. As shown in Fig. 1b, each Cu(II) ion has a square-pyramidal coordination geometry, and is surrounded by two imine N atoms from two different 1,2,4-triazole groups and two O atoms from two different carboxylate groups in the equatorial plane, together with one O atom of the water molecule in the vertex (Supplementary Fig. 1). Topologically, the Cu(II) ion is a planar 4-connected node, further linked by four tetradentate BTTA^2−^ ligands into a three-dimensional network with Kagome-like **USF** topology (Fig. 1d). Consequently, there are hexagonal one-dimensional channels along the *c* direction (Fig. 1c). The pore limiting diameter and the maximum pore diameter predicted by the program Poreblazer^33^ for the fully evacuated **FJI-H14** are 5.95 and 7.62 Å, respectively. The evacuated **FJI-H14** has a theoretical porosity of 44.4% according to PLATON calculations with a probe radius of 1.65 Å. **FJI-H14** has a total concentration of active sites as high as 9.22 mol l^−1^ (the total of Cu(II) OMS is 3.07 mol l^−1^ and free N LBS is 6.15 mol l^−1^), which is higher than in many well-known MOFs for CO_2_ capture (Supplementary Table 2).

### Stability

**Fig. 2:**
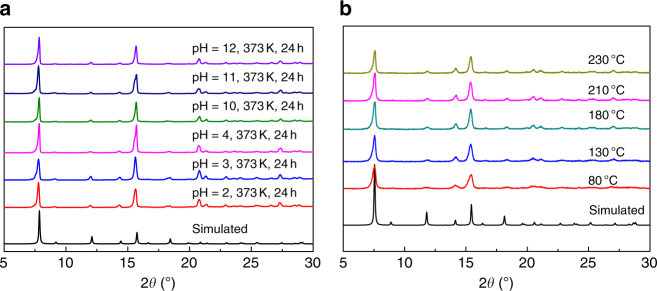
Stability of FJI-H14. **a** PXRD patterns after treatment with boiling water, acid/base environment from pH = 2 to 12 at 373 K. **b** Temperature-dependent PXRD patterns

The flue gas from power plants contains moisture and acid gas, suggesting that the practical adsorbents of post-combustion CO_2_ should be sufficiently stable toward heat, water and acid. This led us to investigate both the chemical stability and thermal stability of **FJI-H14** before the CO_2_ adsorption test. Powder X-ray diffraction pattern (PXRD) analyses reveal that **FJI-H14** is very stable not only in boiling water but also in both acid and base environments at pH = 2 to pH = 12 and at temperatures as high as 373 K (Fig. 2a). However, the framework of **FJI-H14** collapses when it is immersed for 24 h in solution at pH = 1 or pH = 13 (Supplementary Fig. 2). Thermogravimetric analysis (TGA) studies (Supplementary Fig. 3) indicate that the as-synthesized **FJI-H14** sample is thermally stable up to 230 °C, and this is confirmed by temperature-dependent PXRD studies (Fig. 2b and Supplementary Fig. 4). Generally, MOFs based on Cu ions and organic carboxylates are usually subject to hydrolysis in the presence of moisture and only a few known MOFs show such excellent chemical stability^16, 34–38^. The unusual chemical stability of **FJI-H14** may result from its unique structure because the penta-coordinated Cu(II) ion subunit should be more stable than traditional paddle-wheel structure due to the Cu–N coordination interaction, which is stronger than the Cu–O interaction. Furthermore, the abundant free N atoms could also prevent the destruction caused by acids.

### Porosity and CO_2_ adsorption capacity

**Fig. 3:**
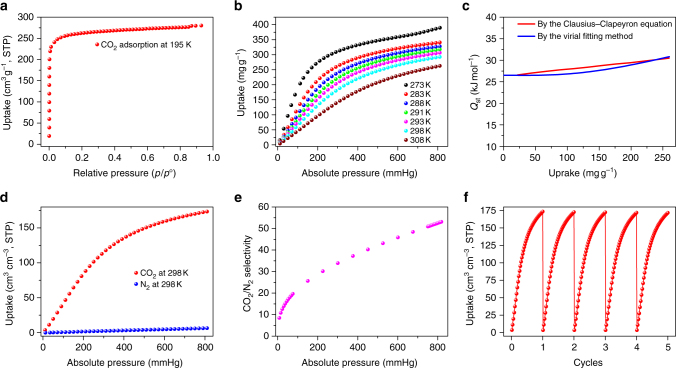
Experimental CO_2_ adsorption by FJI-H14. **a** CO_2_ adsorption isotherm for **FJI-H14** at 195 K. **b** CO_2_ adsorption isotherm for **FJI-H14** at 273, 283, 288, 291, 293, 298 and 308 K. **c** The isosteric heat of CO_2_ adsorption (*Q*_st_) for **FJI-H14** calculated by the Clausius–Clapeyron equation and the Virial fitting method. **d** N_2_ and CO_2_ adsorption isotherms for **FJI-H14** at 298 K. **e** CO_2_/N_2_ selectivity for the 15/85 CO_2_/N_2_ mixture at 298 K. **f** Cycles of CO_2_ adsorption for **FJI-H14** at 298 K

The **FJI-H14** sample for adsorption testing was pre-activated under dynamic vacuum at 100 °C for 10 h after exchanged by acetone for 3 days. PXRD data displayed that the crystallinity was retained after activation (Supplementary Fig. 5). N_2_ adsorption at 77 K was much lower than expected (Supplementary Fig. 6), and consequently, the porosity of activated **FJI-H14** was examined by CO_2_ adsorption experiments at 195 K. A CO_2_ uptake of 279 cm^3^ g^−1^ was obtained (Fig. 3a), corresponding to a formula [Cu(BTTA)]_n_·4.5nCO_2_. A phenomenon that restricted N_2_ uptake at 77 K but supported type-I CO_2_ uptake at 195 K has been observed in several reported MOF materials (Supplementary Table 3). However, **FJI-H14** has a low N_2_ uptake of 170 cm_3_ g^−1^ at 77 K and 1 atm, which is different from the reported MOFs which exhibit almost zero N_2_ adsorption at 77 K. The much lower N_2_ adsorption at 77 K of **FJI-H14** may be a consequence of the relatively narrow pores in **FJI-H14** being easily blocked by the relatively large N_2_ molecule at 77 K, so hindering further diffusion of N_2_ into the framework of **FJI-H14**. The typical type-I isotherm observed indicates that only micropores are formed in the framework of activated **FJI-H14**. The Brunauer–Emmett–Teller (BET) -specific surface area of **FJI-H14** is calculated to be 904 m^2^ g^−1^ and its Langmuir-specific surface area is 1004 m^2^ g^−1^. The total pore volume estimated from the experimental CO_2_ isotherm is 0.45 cm^3^ g^−1^ at P/P_0_ = 0.92, which is slightly higher than the theoretical value of 0.39 cm^3^ g^−1^ derived from the solvent accessible volume and the crystal density through PLATON calculations with a probe radius of 1.65 Å. The comparable values of the pore volume indicate that the activated **FJI-H14** remains permanently porous.

The incorporation of high porosity and the high concentration of open active sites in the framework are expected to lead to high CO_2_ uptake. Indeed, activated **FJI-H14** exhibits remarkable CO_2_ volumetric adsorption capacities of 171 cm^3^ cm^−3^ at 298 K and 1 atm (Fig. 3d), and the capacity is only lower than that of MAF-X25ox (203 cm^3^ cm^−3^)^36^, MAF-X27ox (196 cm^3^ cm^−3^)^36^ and Co_2_(dobdc) (184 cm^3^ cm^−3^) under the same conditions^36, 39^, exceeding that in almost all well-known MOFs such as Mg-MOF-74 (162 cm^3^ cm^−3^)^6, 40^, UTSA-16 (160 cm^3^ cm^−3^)^6, 41^, SIFSIX-2-Cu-i (151 cm^3^ cm^−3^)^42^, MPM-1-TIFSIX (115.7 cm^3^ cm^−3^)^43^, Bio-MOF-11(113 cm^3^ cm^−3^)^6, 44^, Cu-tdpat(103 cm^3^ cm^−3^)^6, 45^ and Mmen−CuBTTri (83 cm^3^ cm^−3^)^6, 30^ (Supplementary Table 2). The formula of CO_2_ adsorbed **FJI-H14** at room temperature is [Cu(BTTA)]_*n*_·2.4nCO_2_ and there is a 53% CO_2_ occupancy at room temperature compared with the maximal uptake at 195 K, which is rather high compared with reported MOFs such as SMT-1 (27%)^46^, [Cu(L)] (31%)^47^ and MPM-1-Cl (29%)^43^. It should be noted that although volumetric adsorption capacity is more practical for stationary CO_2_ capture and separation applications, the gravimetric adsorption capacity is also an important parameter for CO_2_ capture. Although the gravimetric CO_2_ uptake of **FJI-H14** (146 cm^3^ g^−1^) is lower than that of Mg-MOF-74 (176 cm^3^ g^−1^) due to the considerably lighter weight of Mg^40^, it can be comparable to that in other familiar MOF materials such as MAF-X25ox (160 cm^3^ g^−1^)^36^, [Co_2_(dobdc)] (154 cm^3^ g^−1^)^39^ or MAF-X27ox (150 cm^3^ g^−1^)^36^. Another challenging issue is the uptake of CO_2_ at low pressure, which can be highly improved by chemisorption due to the stronger interactions. For instance, the hydrazine functionalized MOF [Mg_2_(dobdc)(N_2_H_4_)_1.8_] (137 cm^3^ cm^−3^) developed by Zhang et al. shows the highest volumetric CO_2_ adsorption capacities at 298 K and 0.15 bar^31^, and the second highest is MAF-X27ox (124 cm^3^ cm^−3^)^36^, which was also prepared by Zhang et al. Based on physisorption, **FJI-H14** displays a volumetric capacity of 60 cm^3^ cm^−3^ at 298 K and 0.15 atm, which makes it comparable to SIFSIX-2-Cu-i (63 cm^3^ cm^−3^). In order to evaluate the affinity of the pore surface of activated **FJI-H14** toward CO_2_, the isosteric heat of adsorption (*Q*_st_) of activated **FJI-H14** was calculated using the Clausius–Clapeyron equation based on the CO_2_ isotherms at seven different temperatures without data fitting (Fig. 3b, Supplementary Fig. 7 and Supplementary Table 4). As shown in Fig. 3c, the *Q*_st_ at low coverage is 26.6 kJ mol^−1^ and then slightly increases to 30.5 kJ mol^−1^ with CO_2_ loading increasing to 260 mg g^−1^. The *Q*_st_ with an increasing slope is unusual^9, 36, 43, 48^, and reveals the possible formation of CO_2_ clusters inside the pores. Such phenomena have been previously observed in other porous MOFs materials^41, 49^. The *Q*_st_, confirmed by the Virial fitting method^50^, also slowly increases from 26.5 to 30.8 kJ mol^−1^ with increasing CO_2_ loading from lower coverage to 260 mg g^−1^ (Fig. 3c and Supplementary Fig. 8). Such similar trends in the two methods confirm the unusual increasing slope in *Q*_st_.

Because flue gas from power plants contains a large amount of N_2_, the CO_2_/N_2_ selectivity is a crucial parameter in CO_2_ capture applications. For comparison, N_2_ sorption isotherms were also measured at 298 K, and showed an uptake of 6.5 cm^3^ cm^−3^ at 1 atm (Fig. 3d). By the ideal adsorbed solution theory (IAST)^51^, the CO_2_/N_2_ selectivity for the 15/85 CO_2_/N_2_ mixture at 1 atm is calculated to be 51 at 298 K (Fig. 3e). The highly selective adsorption of CO_2_ over N_2_ further suggests that the densely populated open active sites in the framework have a positive effect on CO_2_ adsorption. The possibility of reuse of an adsorbent is also an important aspect of the practical application. Further research demonstrates that activated **FJI-H14** can be recycled without losing its adsorption capacity. Even after five cycles, it still maintains 100% adsorption capacity as shown in Fig. 3f, indicating that **FJI-H14** is highly suitable for CO_2_ capture.

### Revealing the CO_2_ adsorption sites in FJI-H14

**Fig. 4:**
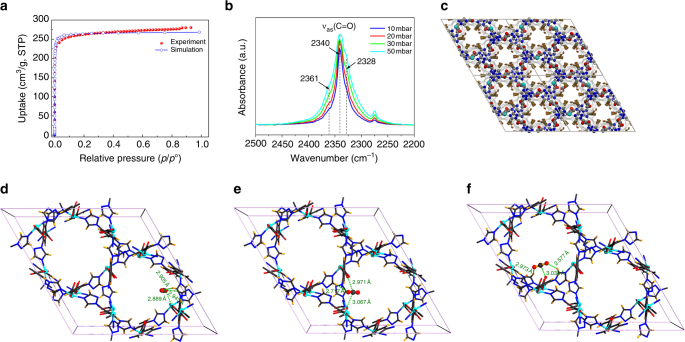
Mechanism of CO_2_ adsorption of FJI-H14. **a** Experimental and simulated excess CO_2_ adsorption isotherms at 195 K. **b** Background-subtracted FTIR spectra of CO_2_ adsorbed on **FJI-H14** at decreasing equilibrium pressure. **c** Density picture of adsorbed CO_2_ (1 CO_2_ at 273 K), which is shown as a volume rendered mode. **d**–**f** represent three preferential CO_2_ locations in **FJI-14** obtained from GCMC simulation and DFT optimization

The extraordinary CO_2_ capture performance of **FJI-H14** under ambient conditions has motivated us to rationalize the crucial factors supporting the high CO_2_ adsorption capacity. To understand the sorption behavior of **FJI-H14**, both grand canonical Monte Carlo (GCMC) simulations and density functional theory (DFT) calculations have been carried out and are shown in Fig. 4. The GCMC simulations show that the theoretical CO_2_ adsorption isotherms of **FJI-H14** are basically consistent with the experimental data at different temperatures (195, 273 and 298 K) (Fig. 4a, Supplementary Fig. 9). The simulated CO_2_ concentration loaded into **FJI-H14** is about 4.3, 3.1 and 2.5 CO_2_ per ligand molecule at 195, 273 and 298 K, respectively. These figures are close to the experimental values of 4.5, 3.1 and 2.4 CO_2_ per ligand molecule at 195, 273 and 298 K. As shown in Fig. 4c, the adsorbed CO_2_ molecules at low coverage prefer to locate the corners of hexagonal channels. Combining the density plots and a snapshot of the adsorbed CO_2_ molecules, three typical preferential CO_2_ adsorption sites can be observed. These three adsorption sites were further optimized by DFT methods using the Dmol^3^ module. Site I (Fig. 4d) shows that CO_2_ molecules prefer to coordinate with open Cu(II) ions through Cu–O interaction, with a Cu–O distance of about 2.889 Å, and each copper site binding only one CO_2_^52^. In Site II (Fig. 4e), the two closest C–O distances are about 2.971 and 3.067 Å, indicating that O atoms of carboxyl group could also interact as a Lewis base with CO_2_. It is also interesting to find that positively charged H atoms could further promote CO_2_ adsorption, with the shortest O-H distances about 2.577 Å as shown in Site III (Fig. 4f). The calculated static CO_2_ binding energy of the above three different preferential CO_2_ adsorption sites are ~43.71, 38.94, 32.82 kJ mol^−1^, respectively, indicating that the open copper sites could play a leading role in CO_2_ adsorption.

**Fig. 5:**
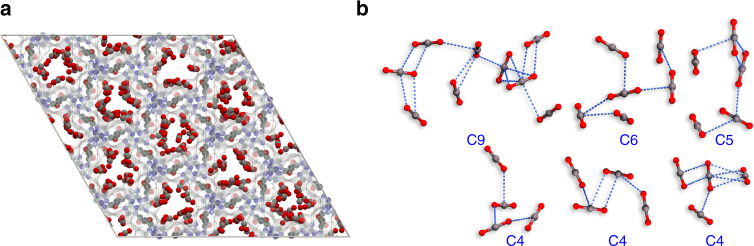
The snapshot for CO_2_-loaded FJI-H14. **a** The snapshot of CO_2_ adsorption for **FJI-H14** at 11.2 kPa and 273.15 K calculated using the GCMC method. **b** The representative CO_2_ clusters including nine (C9), six (C6), five (C5) and four (C4) CO_2_ molecules. The blue dashed line represents weak interactions between neighboring CO_2_ molecules with a short C···O separation (from 2.71 to 3.50 Å) for adjacent CO_2_ molecules

The simulated *Q*_st_ from the GCMC simulations can be used to deduce the information on *Q*_st_ with an increasing slope for **FJI-H14**. As shown in Supplementary Fig. 10, the simulated *Q*_st_ of 30.7–34.6 kJ mol^−1^ is slightly larger than suggested by experimental results and has a uniformly increasing trend with increasing CO_2_ loading. The contribution of the CO_2_···Framework interaction to the total *Q*_st_ decreases slightly with increasing loading, which is reasonable because CO_2_ molecules first occupied the more active sites. However, the contribution from the CO_2_···CO_2_ interactions shows a tendency to increase significantly from 0 to 7.0 kJ mol^−1^, which is due to the closer packing of the CO_2_ molecules under the higher pressure in the relatively narrow pores. Hence, the behavior of the total *Q*_st_ is the result of the two cooperative contributions. The increasing contribution from the CO_2_···CO_2_ interactions indicates that the CO_2_ clusters could have formed inside the pores. Hence, GCMC simulation was used to investigate the potential CO_2_ clusters. Some small CO_2_ clusters can be found in the snapshot at 195 K and low pressure (~ 21 Pa) calculated using the GCMC method (Supplementary Fig. 11), but at 273 K, similar CO_2_ clusters can only be observed under a relatively high pressure of 11 kPa. The snapshots of the framework of **FJI-H14** with CO_2_ molecules adsorbed are shown in Fig. 5a. The weak interactions between neighboring CO_2_ molecules are found in terms of the short C···O separation (from 2.71 to 3.50 Å) for adjacent CO_2_ molecules, which links those CO_2_ molecules into small clusters (Fig. 5b and Supplementary Fig. 12).

To verify the preferential adsorption sites experimentally, in situ fourier transform infrared microscope (FT-IR) spectra of activated **FJI-H14** sample have been collected at increasing equilibrium pressure under a CO_2_ atmosphere. Figure 4b shows the background-subtracted IR spectra obtained by the progressive lowering of equilibrium pressure at room temperature. The strong absorption bands at 2,340 and 2,328 cm^−1^ red-shifted by Δ*ν* of about −9 and −21 cm^−1^ from gas phase CO_2_ asymmetric stretch (*ν*_as_ = 2349 cm^−1^) might be attributed to the *ν*_as_ mode of CO_2_ (Supplementary Fig. 13) interacting with Cu(II) centers. The slightly blue-shifted band at 2,361 cm^−1^ (Δ*ν* =  + 12 cm^−1^ shift) can be readily assigned to the asymmetric *ν*_as_ stretch of CO_2_ interacting with the exposed Lewis base sites throughout the channel. On the low-frequency side of this main absorption, the less intense bands at 2,275 cm^−1^ result from the interaction between Cu(II) center and ^13^CO_2_ which is present (1%) naturally in ^12^CO_2_. The stronger absorption bands at 2,340 cm^−1^ and 2,328 cm^−1^ also indicate that CO_2_ molecules tend to stack around the open Cu(II) sites, which is in accord with the above theoretical calculation.

### Large-scale synthesis

**Fig. 6:**
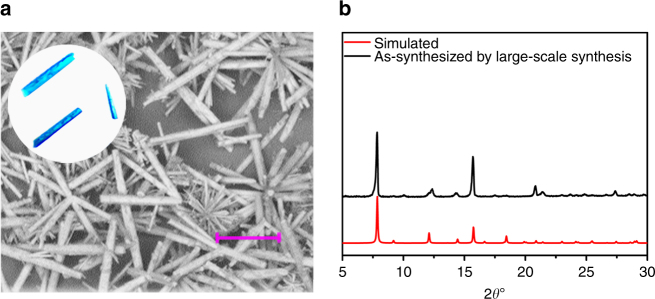
Large-scale synthesis of FJI-H14 microcrystals. **a** Morphology comparison between SEM image of **FJI-H14** microcrystals and of **FJI-H14** single crystals (inset). Scale bars, 10 µm. **b** PXRD patterns comparison: black represents simulated **FJI-H14**; red represents **FJI-H14** microcrystals

For practical applications, efficient macroscopic preparation and purification are a bottleneck problem which must be solved. After many attempts, the following protocol for large-scale preparation of **FJI-H14** has been established: by directly mixing H_2_BTTA ligand and Cu(NO_3_)_2_ in water and then refluxing for 1 day, microcrystalline **FJI-H14** can be obtained with a high yield of 90%, its purity confirmed by PXRD analysis (Fig. 6b). Scanning electron microscopy measurements demonstrate that the relatively uniform rod crystallites form on the scale of about 20 μm, displaying a similar morphology with the single crystal (Fig. 6a) obtained from the hydrothermal reaction. Therefore, macroscopic samples of **FJI-H14** even on a 10 g scale can be readily synthesized by this method, which makes its application more possible.

### Catalyzed cycloaddition of CO_2_ using flue gas as feedstock

Another attractive means of effective elimination of CO_2_ is the direct chemical conversion of CO_2_ into value-added chemicals, such as dimethyl carbonate, cyclic carbonates, *N*,*N*’-disubstituted ureas or formic acid. Given their wide applications in the pharmaceutical and fine chemical industries, cyclic carbonates formed by the coupling of epoxides with CO_2_ have attracted intense interest. Although several existing MOFs have been shown to be excellent heterogeneous Lewis acid catalysts for chemical conversion of CO_2_ either at high pressure or normal pressure^53–56^, the exploration of practically useful MOF materials which can catalyze the conversion of CO_2_ obtained directly from power plants remains a challenge to be addressed. The high density of OMSs and LBSs which are finely distributed throughout the channel pores of **FJI-H14**, are devoted to capturing CO_2_ effectively and selectively under ambient conditions. Such unusual synergistic effects may also improve chemical conversion of post-combustion CO_2_ from the power plant. Here, we use a mixed gas which contains 0.15 atm CO_2_ and 0.85 atm N_2_ to simulate flue gas from the power plant. As expected, **FJI-H14** displays a much higher catalytic activity for cycloaddition of styrene oxide with the simulated flue gas than other catalysts, such as homogeneous Cu(OAc)_2_, heterogeneous HKUST-1 and a mixture of Cu(NO_3_)_2_ and free H_2_BTTA ligand. As shown in Table 1, absence of extra Lewis acid catalysts only lead to a moderate yield (52%), while use of extra Cu(II) catalyst can improve their reaction activity, with the exception of Cu(OAc)_2_. Catalyzed by **FJI-H14**, chemical conversion of diluted CO_2_ with styrene oxide into corresponding cyclic carbonates gives a yield of 86% within 24 h, while use of homogeneous Cu(OAc)_2_ and the mixture of Cu(NO_3_)_2_ and H_2_BTTA leads only to 45 and 70% yields respectively under the same reaction conditions. Further tests demonstrate that the **FJI-H14** is more active than well-known HKUST-1 which leads to only 67% yield under the same conditions. All these results indicate that **FJI-H14** is indeed an efficient catalyst for chemical conversion of CO_2_ under practical conditions. In order to investigate the catalytic character of **FJIH-14**, another two different sized substrates have been selected. As shown in Table 1 entries 5–6, the smaller (chloromethyl)ethylene oxide gives a higher yield (95%), while 1,2-epoxyoctane leads to a lower yield (27%), indicating that the cycloaddition reaction may occur within the pores of **FIJ-H14**, into which smaller-sized substrates could easily diffuse and make contact with the active sites. To further prove that the reaction may occur in the pores of **FJI-H14**, uptake of different reactants has also been assessed. Further analysis demonstrates that (chloromethyl)ethylene oxide indeed diffuses into the pores of **FJI-H14** more easily than 1,2-epoxyoctane and styrene oxide which apparently have similar diffusion rates. Considering the similar diffusion from 1,2-epoxyoctane and styrene oxide, the higher activity of styrene oxide compared to 1,2-epoxyoctane may result from following two factors: first, a phenyl ring is an electron-withdrawing group, which can improve reaction activity, and second, the *π–π* interaction from the phenyl ring of styrene oxide and the aromatic rings of the **FJI-H14** framework also can improve reaction activity. This may provide a strategy for development of more practical catalysts for the conversion of CO_2_ directly from flue gas.

**Table 1:** Cyclic carbonates from epoxides and CO_2_^a^

	

^a^Reaction conditions: styrene oxide (20.0 mmol), catalyst (0.48 mol% per Cu(II) units), TBAB (2.5 mol%) in a Schleck tube with condenser, 1 atm simulated post-combustion flue (CO_2_ = 0.15 atm, N_2_ = 0.85 atm) was bubbled at 80 °C for 24 h

^b^Determined by ^1^H NMR.

^c^Some by-products were found when Cu(OAc)_2_ was used as a catalyst

## Discussion

Considering that most of CO_2_ emission is generated from power stations, direct elimination of such CO_2_ should play an important role in the reduction of global CO_2_ emissions. The characteristics and composition of post-combustion CO_2_ determine that an ideal adsorbent for post-combustion CO_2_ capture should possess advantages such as high CO_2_ uptake and selectivity at ambient pressure, excellent chemical stability and thermal stability, good reusability and large-scale production with low cost. Porous MOFs have been proved to be effective adsorbents for CO_2_ capture due to their large capacity for the adsorption of gases, but development of an ideal MOF for post-combustion CO_2_ capture is still challenging. Although many different OMS and LBS have been introduced into MOFs to improve CO_2_ capture, only very few porous MOFs have been realized for high CO_2_ capture at ambient conditions, and most of them are sensitive to water. **FJI-H14** not only shows extraordinary high volumetric uptake of CO_2_ with high selectivity under ambient conditions but is also highly resistant to water and an acid/base environment; furthermore, it also can be reused without loss of adsorption capacity and prepared on a large-scale with low cost. These advantages make **FJI-H14** an ideal and practical adsorbent for post-combustion CO_2_. An unusual synergistic effect from multiple active sites has also been observed, and may provide a strategy for the design of more effective adsorbents for CO_2_ capture. Further chemical conversion of captured CO_2_ to high-value products, such as cyclic carbonate, is also attractive, and several existing MOFs have been proved to be excellent heterogeneous Lewis acid catalysts for chemical conversion of pure CO_2_. However, development of MOF materials which can catalyze the direct conversion of post-combustion CO_2_ still remains a challenge. It is shown here that **FJI-H14** can directly and smoothly catalyze the chemical transformation of simulated post-combustion gas CO_2_ into corresponding cyclic carbonates. All these results should be instructive for the design and discovery of more effective and practical MOF materials for the elimination of post-combustion CO_2_ in the near future.

## Methods

### Synthesis and scale up

A mixture of Cu(NO_3_)_2_·3H_2_O (0.05 mmol, 12 mg) and H_2_BTTA (0.05 mmol, 15 mg) in H_2_O (4 ml) was sealed in a 23 mL Teflon vial, which was heated at 120 °C for 3 days, then cooled to room temperature. After washing with fresh acetone, blue crystals of **FJI-H14** were obtained in 73% yield based on the organic ligand H_2_BTTA. Elemental analysis was calculated for **FJI-H14**: C, 29.54%; H, 4.13%; N, 17.23%. Found: C, 29.35%; H, 4.12%; N, 17.29%. For scale up, a mixture of Cu(NO_3_)_2_·3H_2_O (1 mmol, 241.6 mg) and H_2_BTTA (1 mmol, 300.1 mg) in H_2_O (80 ml) was refluxed for 1 day, and then the blue powder of **FJI-H14** microcrystals could be obtained in 90% yield based on H_2_BTTA. After three washings with water and two with acetone, the phase purity of the sample was confirmed by PXRD.

### Characterization

Elemental analyses for C, H, N were carried out on a German Elementary Vario EL III instrument. The ^1^H NMR spectra were measured on an AVANCE III Bruker Biospin spectrometer, operating at 400 MHz. Thermogravimetric analyses (TGA) were recorded on an NETZSCH STA 449 C unit at a heating rate of 10 °C min^−1^ under flowing nitrogen atmosphere. In situ FT-IR spectra were obtained using a NICOLET 6700 instrument at 298 K. The PXRD patterns were collected using a Rigaku MiniFlex 600 X-ray diffractometer with monochromatic Cu Kα radiation (*λ* = 1.54 Å). Simulations of the PXRD spectrum were carried out by the single-crystal data and diffraction-crystal module of the Mercury program, available free of charge via the internet at https://www.ccdc.cam.ac.uk/solutions/csd-system/components/mercury/.

### Single-crystal X-ray diffraction

The single-crystal data of **FJI-H14** was collected on a SuperNova diffractometer at 100 K. The structure was solved using *SHELXT*-2014 and refined by full-matrix least squares on *F* ^2^ with *SHELXL*-2014^57^. All the non-hydrogen atoms were refined anisotropically. Hydrogen atoms of the organic ligands were generated theoretically onto the specific atoms and refined isotropically. We employed *PLATON/SQUEEZE* ^58^ to calculate the contribution to the diffraction from the solvent region and thereby produced a set of solvent-free diffraction intensities. The final formula was calculated from the SQUEEZE results combined with elemental analysis data and TGA data. Crystallographic data and structure refinement parameters for this crystal are summarized in Supplementary Table 1.

### Gas-adsorption

Low-pressure (<1 bar) adsorption measurements were performed using an Accelerated Surface Area and Porosimetry 2020-M System. Before the measurements, about 100 mg solvent-exchanged samples were loaded into the sample tube and then degassed under dynamic vacuum at 100 °C for 10 h to obtain fully desolvated samples. Low-pressure N_2_ adsorption isotherms were measured at 77 K in a liquid nitrogen bath (Supplementary Fig. 6). Low-pressure CO_2_ adsorption isotherms were measured at 195, 273, 283, 288, 291, 293, 298 and 308 K. The specific surface areas were determined using the BET model from the CO_2_ adsorption isotherm.

### The isosteric heat of adsorption

Method 1: the isosteric heat of adsorption Q_st_ was calculated using the Clausius–Clapeyron equation (equation (1)).

1 $$\ln \left( {{P_i}} \right) = {Q_{st}} \times \frac{1}{{{\rm{R}}{T_i}}} + {\rm{C,}}$$

where *P*_i_ is the pressure of the isotherm *i* (kPa), *T*_i_ is the temperature of isotherm *i* (K), R is the gas constant and C is a constant. The Qst is subsequently obtained from the slope of plots of ln(*P*_i_) as a function of 1/T (Supplementary Fig. 7 and Supplementary Table 4).

Method 2: the Qst was estimated from isotherms at different temperatures applying the Virial fitting method (equation (2) and Supplementary Fig. 8). The fitting parameters were then used to calculate the *Q*_st_ using equation (3).

2 $${\rm{ln}}\,P = {\rm{ln}}\,N + \frac{1}{T}\mathop {\sum}\limits_{i = 0}^m {{a_i}{N^i}} + \mathop {\sum}\limits_{i = 0}^n {{b_i}{N^i}} ,$$

3 $${Q_{{\rm{st}}}} = - R\mathop {\sum}\limits_{i = 0}^m {{a_i}{N^i}} ,$$

where *P* is the pressure (mmHg), *N* is the adsorbed quantity (mg g^−1^), *T* is the temperature (K), *R* is the gas constant, *a*_i_ and *b*_i_ are virial coefficients and *m* and *n* represent the number of coefficients required to adequately describe the isotherms (herein, *m* = 5, *n* = 2).

### Calculation of gas selectivity based on IAST

The gas adsorption isotherms were first fitted to a Langmuir-Freundlich model. IAST starts from the Raoults’ Law type of relationship between fluid and adsorbed phase.

4 $${P_i} = P{y_i} = P_i^o{x_i},$$

5 $$\mathop {\sum}\limits_{i = 1}^n {{x_i}} = \mathop {\sum}\limits_{i = 1}^n {\frac{{{P_i}}}{{P_i^0}}} = 1,$$

where *P*_*i*_ is the partial pressure of component *i* (kPa), *P* is the total pressure (kPa), *y*_*i*_ and *x*_*i*_ represent mole fractions of component *i* in gas and adsorbed phase (dimensionless). $P_i^0$ is the equilibrium vapor pressure (kPa).

In IAST, $P_i^0$ is defined by relating to spreading pressure π,

6 $$\frac{{\pi S}}{{RT}} = \mathop {\int}\limits_0^{P_i^0} {\frac{{{q_i}({P_i})}}{{{P_i}}}d{P_i}} = \Pi \,\left( {{\rm{Constant}}} \right),$$

where *π* is the spreading pressure, *S* is the specific surface area of adsorbent (m^2^ g^−1^), *R* is the gas constant, *T* is the temperature (K) and *q*_*i*_(*P*_*i*_) is the single component equilibrium obtained from the isotherm (mg g^−1^).

The isotherm parameters are known from the previous fitting. The adsorption selectivities *S*_ads_ were calculated using equation (7).

7 $${S_{ads}} = \frac{{{q_1}/{q_2}}}{{{p_1}/{p_2}}}.$$

In this study, IAST calculations were carried out assuming CO_2_/N_2_ (15/85) binary mixed gases at 298 K and pressure up to 1 bar to mimic the composition and condition of flue gas for post-combustion CO_2_ capture.

### Computational methodologies

The GCMC simulations for CO_2_ at 195, 273 and 298 K and up to 100 kPa were performed using with *RASPA* v2.03^59^. The **FJI-H14s** framework was generated in the *R*3 space group based on the crystallographic data of **FJI-H14** to avoid disorder in the structure (Supplementary Fig. 14). Twelve unit cells of **FJI-H14s** (2 × 2 × 3) were used to construct the simulation box of the GCMC run. The structural parameters of simulation box are *a* = *b* = 44.9714 Å and *c* = 33.1527 Å, as well as *α* = *β* = 90° and *γ* = 120°. The partial charges on the framework atoms were calculated by the Gaussian09 software at the B3LYP/6–31 G* level of theory^60^. Partial atomic charges were extracted using the ChelpG method by fitting them to reproduce the electrostatic potential generated by the DFT calculations. The charge was adjusted slightly in order to result in a neutral framework. Resulting partial charges for **FJI-H14s** are given in Supplementary Table 5.

CO_2_–CO_2_ and CO_2_-framework interactions were calculated using a Lennard–Jones (LJ) + Coulomb potential. LJ parameters for the framework atoms were taken from the Dreiding Force Field except for the copper atom, for which the parameters were taken from UFF (Supplementary Table 7). The CO_2_ LJ parameters were taken from an empirical TraPPE force field with a partial charge on each atom (Supplementary Table 6). The mixing LJ parameters between different atomic types were calculated according to the Lorentz–Berthelot mixing rule. Lorentz–Berthelot mixing rules were used for all cross terms, and LJ interactions beyond 12.8 Å were neglected. The Ewald sum method was used to compute the electrostatic interactions. The fugacity of CO_2_ was calculated using the Peng-Robinson equation of state with the corresponding parameters. Simulations for CO_2_ adsorption used 100,000 cycles for equilibration and 100,000 cycles for data collection. In a cycle, N Monte Carlo moves were performed, where N is whichever value is larger between 20 and the number of molecules in the system. Monte Carlo moves used with equal probability were translation, rotation, insertion, deletion, and random reinsertion of an existing molecule at a new position, while framework atoms remained fixed at their original positions.

The simulated isosteric heats of adsorption are computed from the GCMC simulations using the expression (equation (8)).

8 $${Q_{st}} = {\rm{R}}T - \frac{{\langle {U_{{\rm{gg}}}}N\rangle - {\langle U_{{\rm{gg}}}\rangle}\langle N\rangle}}{{\langle {N^2}\rangle - {\langle N \rangle ^2}}} - \frac{{{\langle U_{{\rm{gf}}}N\rangle} - {\langle U_{{\rm{gf}}}\rangle} \langle N\rangle}}{{{\langle N^2\rangle} - {\langle N\rangle^2}}},$$

where the brackets 〈 〉 denote the ensemble average, R is the gas constant, *N* is the number of gas molecules in the system, *U*_gg_ is gas···gas interaction energy and *U*_gh_ is the gas···framework interaction energy. The second and third terms are the contributions to the simulated *Q*_st_ from the gas···gas interaction and the gas···framework interaction, respectively.

DFT methods help shed light on adsorption mechanisms by calculating the adsorption energy of CO_2_ in MOF. Three possible main adsorption sites for adsorbed CO_2_ in MOF were investigated by the Dmol^3^ module integrated into the Material Studio 7.0 program package^61^. The PBE-type exchange-correlation functional^62^ with a generalized gradient approximation and the Double Numerical plus polarization (DNP) basis sets^63^ that include a d-type polarization function on all non-hydrogen atoms and a p-type polarization function on all hydrogen atoms were employed for all calculations^64^. The FINE quality mesh size was employed in the calculations. During the CO_2_-MOF structure optimization, the lattice parameters and the atomic fraction positions of the MOF crystal were kept immobile and the single CO_2_ molecule was allowed to move during optimization. The possible adsorption sites are shown in the Fig. 4d–f. The adsorption energies were calculated in terms of equation (9)

9 $${E_{{\rm{ads}}}}{\rm{ = }}{E_{{\rm{MOF}} - {\rm{C}}{{\rm{O}}_2}}} - {E_{{\rm{MOF}}}} - {E_{{\rm{C}}{{\rm{O}}_2}}},$$

where ${E_{{\rm{MOF}} - {\rm{C}}{{\rm{O}}_2}}}$ stands for the energy of the optimized adsorbate-MOF structure, and *E*_MOF_, and E_CO2_ denote the energies of the bare MOF structure and the isolated CO_2_ molecule, respectively. According to this equation, a more negative adsorption energy means more favorable binding.

### Catalyzation of cycloaddition of simulated post-combustion CO_2_

20 mmol styrene oxide, 0.48 mol% per Cu(II) units (for example, 18 mg activated **FJI-H14** (0.016 mmol), 8.7 mg Cu(OAc)_2_ (0.048 mmol)), and 164 mg TBAB (0.5 mmol, 2.5 mol%) were placed in a 5 mL dry Schleck tube with condenser, then 1 atm simulated post-combustion flue gas (CO_2_ = 0.15 atm, N_2_ = 0.85 atm) was introduced by bubbling, and the reaction mixture was stirred at 80 °C for 24 h.

### Uptake of different reactants

10 mg of activated crystals of **FJI-H14** was placed in a dry 5 ml Schleck flask, the flask was then evacuated under dynamic vacuum at 80 °C for 2 h and filled with argon, then 1 ml reactant was injected and the reaction was kept under argon atmosphere. 10 min later, the inclusion crystals of **FJI-H14** were filtered, after removing surface reactant molecules; the inclusion reactants can be readily removed from inclusion crystals **FJI-H14** by ultrasonic processing in DMSO-d^6^ solution and further determined by ^1^H NMR. Finally, about 2.78 μmol of (chloromethyl)ethylene oxide, 1.2 μmol of 1,2-epoxyoctane, or 1.1 μmol of styrene oxide were added.

### Data availability

The X-ray crystallographic coordinates for structure reported in this article have been deposited at the Cambridge Crystallographic Data Centre (CCDC), under deposition number CCDC 1517725. These data can be obtained free of charge from The Cambridge Crystallographic Data Centre via www.ccdc.cam.ac.uk/data_request/cif.

## Electronic supplementary material

Supplementary Information

Description of additional supplementary files

Dataset 1

Dataset 2
