# Supplementary material for: Carbon dioxide capture and conversion by an acid-base resistant metal-organic framework
Source: Nat Commun. 2017 Nov 1;8:1233. doi: 10.1038/s41467-017-01166-3 (PMC5663901; doi:10.1038/s41467-017-01166-3)
Supplement: Supplementary file 1 — Supplementary Information [file 41467_2017_1166_MOESM1_ESM.pdf]

Supplementary Figures

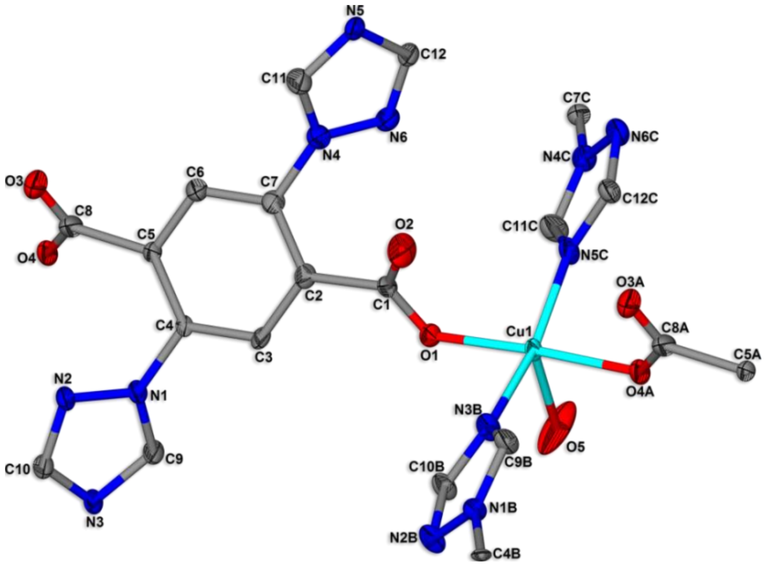

Supplementary Figure 1. Coordination environment of Cu(II) center in **FJI-H14** with H atoms being omitted for clarity (ellipsoids at 35% probability). Cu1–O1 1.963(5) Å, Cu1–O5 2.259(5) Å, Cu1–O4 1.968(7) Å, Cu1–N3 2.004(7) Å, Cu1–N5 2.005(6) Å. Symmetry codes: A: x, y, 1+z; B: 1/3-y, 2/3+x-y, 2/3+z; C: 2/3-y, 1/3+x-y, 1/3+z.

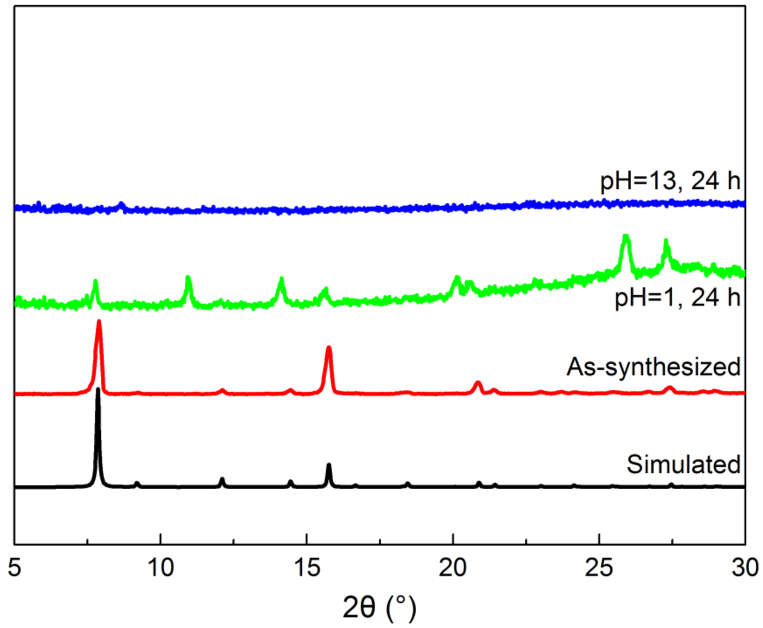

Supplementary Figure 2. PXRD patterns for **FJI-H14** after treatment with water, acid/base environment at pH = 1 and 13 for 24 h.

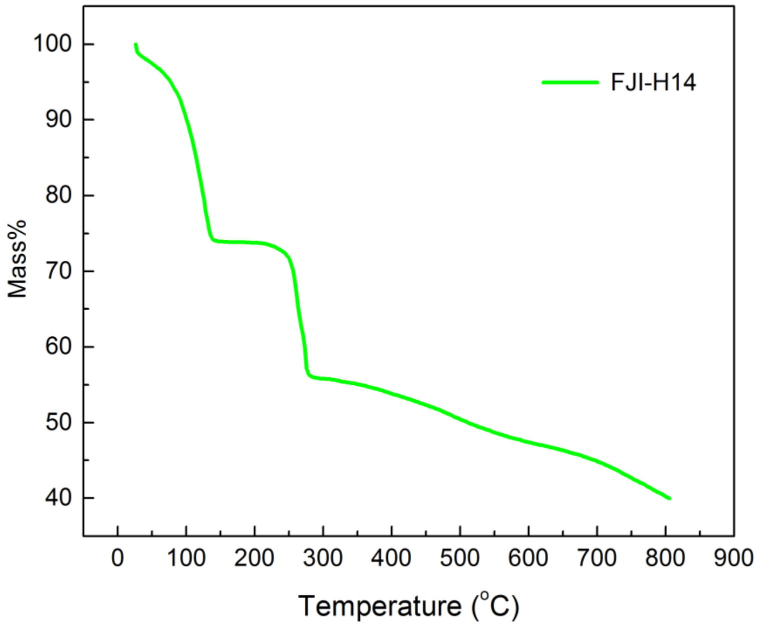

Supplementary Figure 3. TGA curves for **FJI-H14** samples.

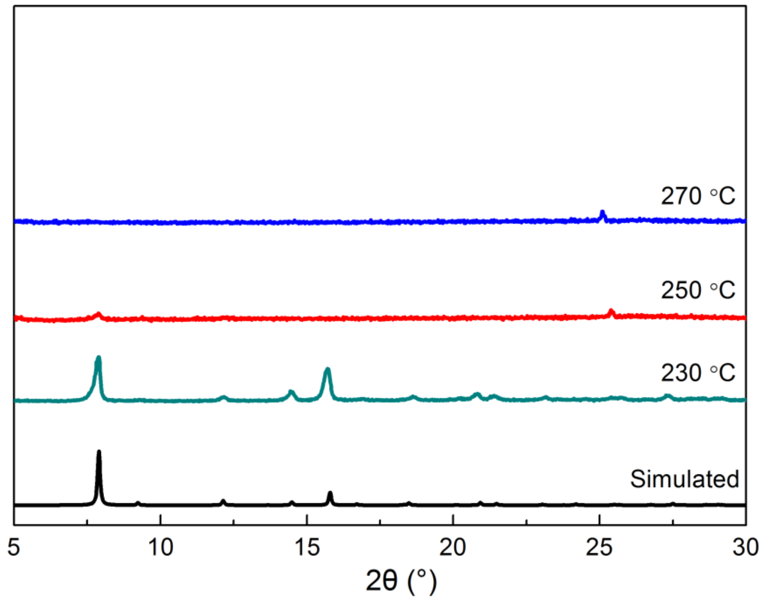

Supplementary Figure 4. PXRD patterns for **FJI-H14** at temperature higher than 230 °C.

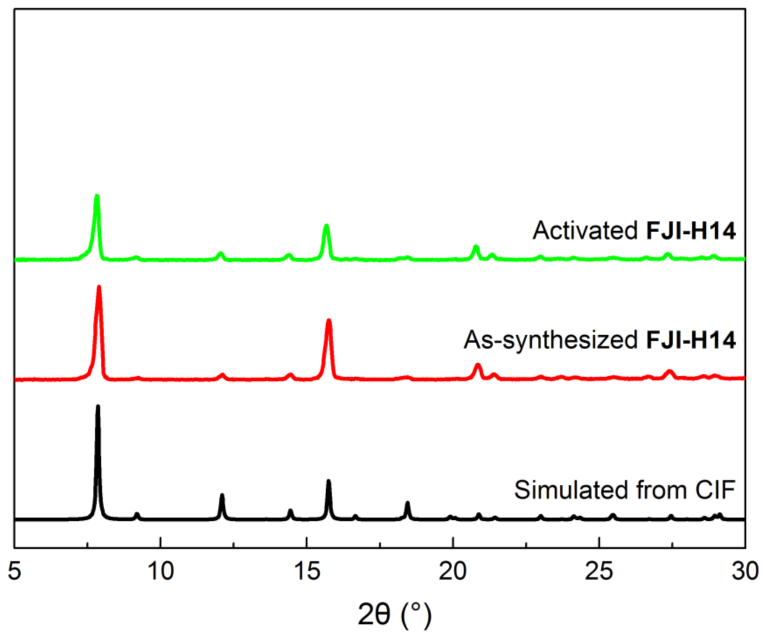

Supplementary Figure 5. Powder X-ray diffraction patterns from: simulated from cif file of **FJI-H14** (black), as-synthesized **FJI-H14** (red) and activated **FJI-H14** (green).

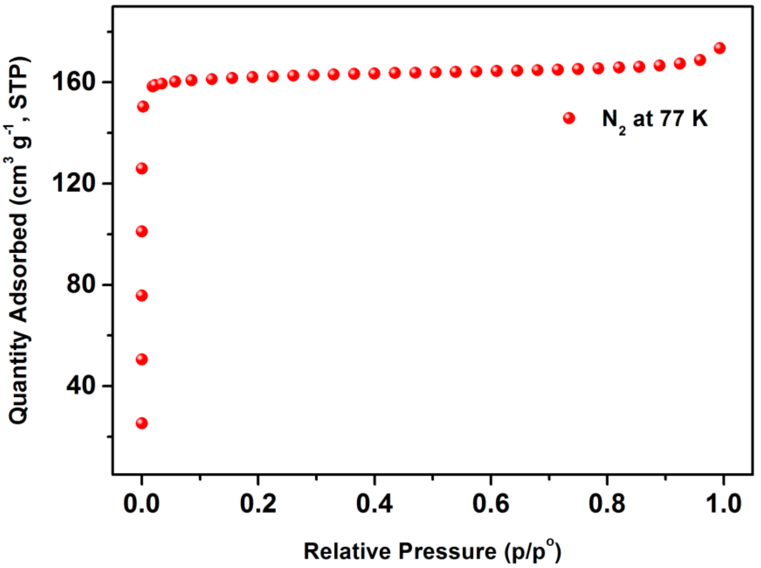

Supplementary Figure 6. N_2 adsorption isotherm at 77 K

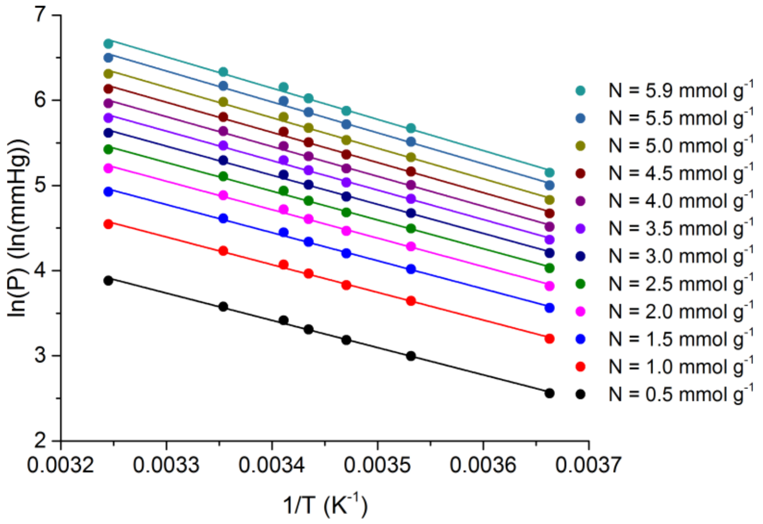

Supplementary Figure 7. Vant' Hoff isochores plot for CO_2 adsorption on **FJI-H14** in the uptake range of 0.5 to 2.9 mmol g^{-1}.

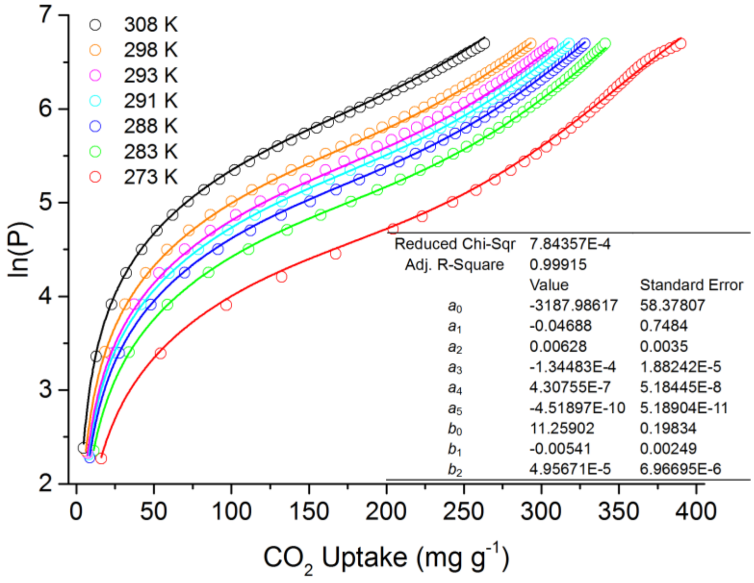

Supplementary Figure 8. The calculated virial equation isotherms parameters fit to the experimental CO_2 data of **FJI-H14**.

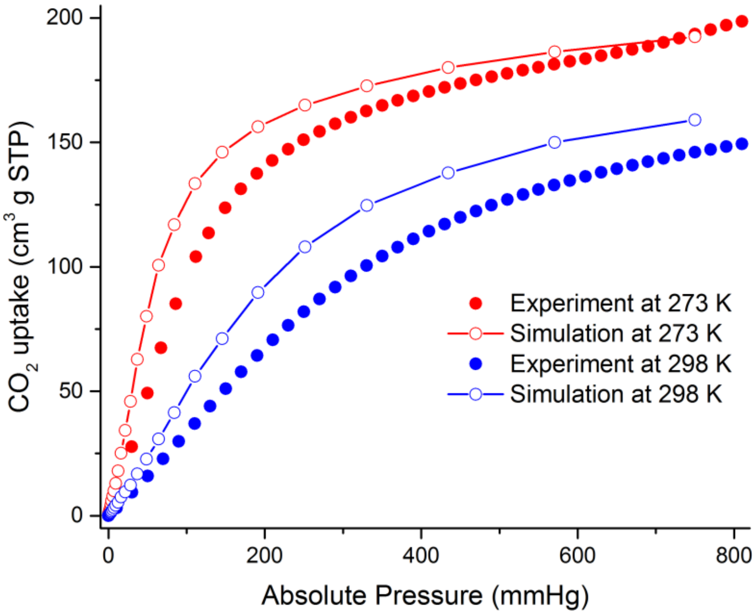

Supplementary Figure 9. Experimental and simulated excess CO_2 adsorption isotherms at 273 K and 298 K

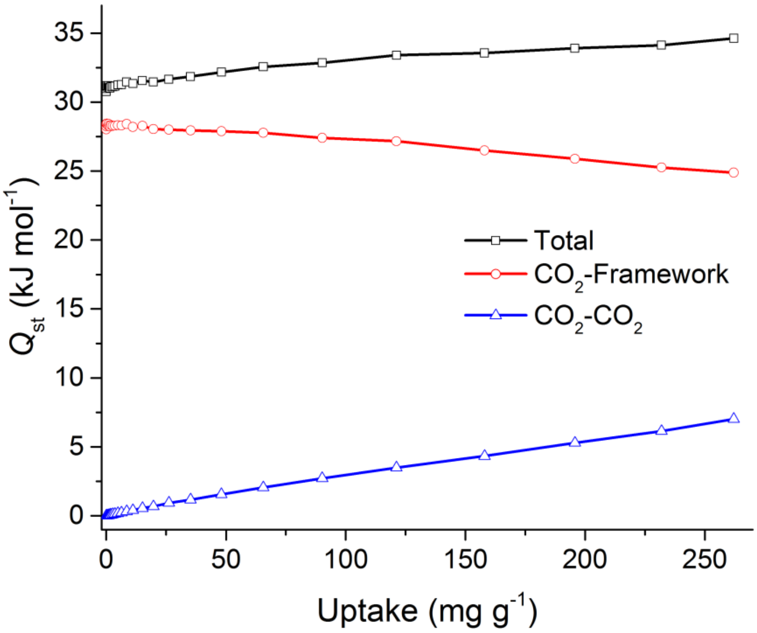

Supplementary Figure 10. Various contributions to simulated heats of adsorption for CO_2 in FJI-H14 at 273 K, as a function of uptake.

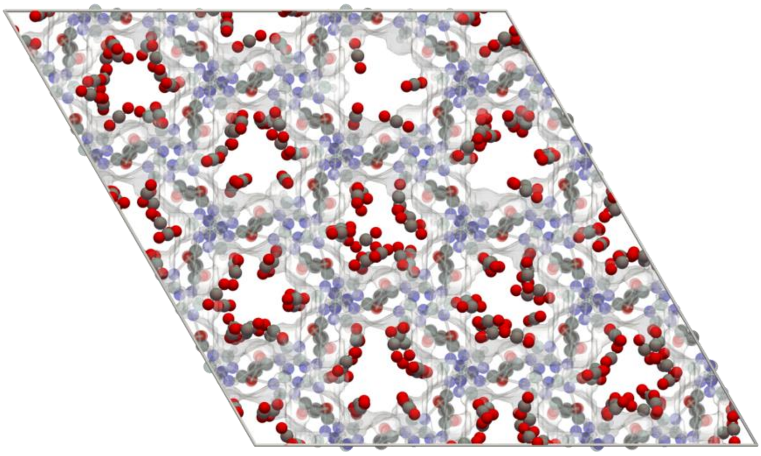

Supplementary Figure 11. Snapshot of CO_2 adsorption for **FJI-H14** at 21 Pa and 195 K calculated using the GCMC method.

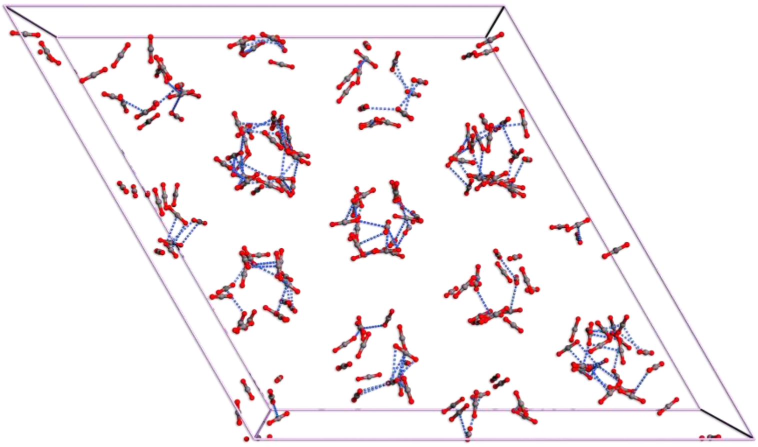

Supplementary Figure 12. The weak interactions among CO_2 molecules adsorbed at pores of **FJI-H14** at 11.2 kPa and 273.15 K calculated using the GCMC method. For clarity, the framework atoms of **FJI-H14** are hided.

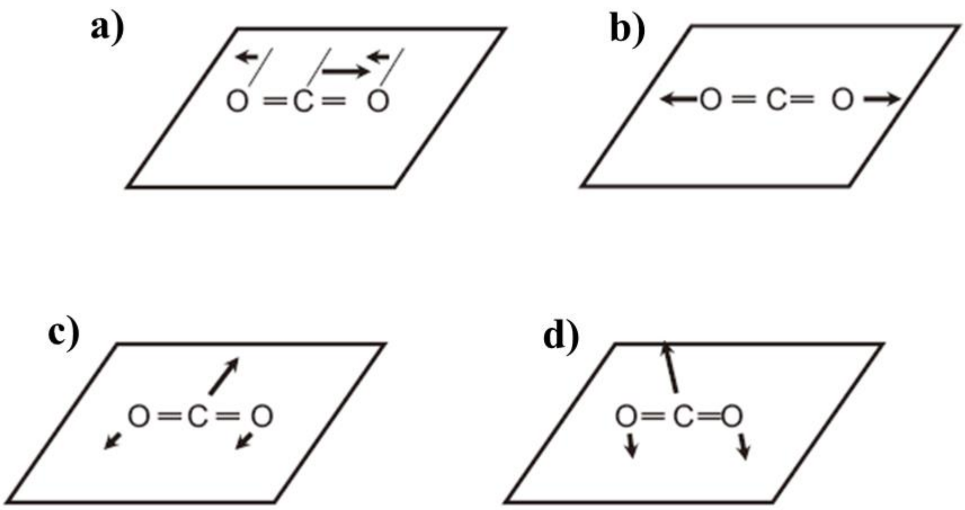

Supplementary Figure 13. a) asymmetric stretching vibration, b) symmetric stretching vibration, c) in-plane bending vibration, d) out-of- plane bending vibration.

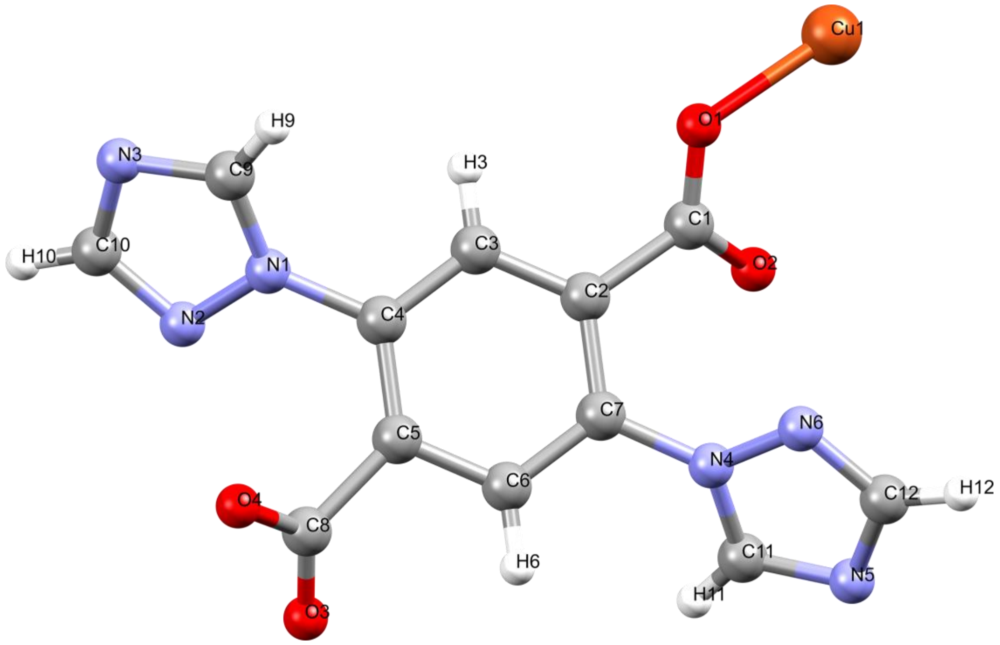

Supplementary Figure 14. The unit used for deriving partial charges on atoms in **FJI-H14**.

Supplementary Table

Supplementary Table 1. Crystal data and structure refinement for **FJI-H14**

**Identification code**	**FJI-H14**
Empirical formula	$C_{36}H_{18}Cu_3N_{18}O_{15}$
Formula weight	1133.30
Temperature	100.0(4) K
Wavelength	1.54184 Å
Crystal system	Trigonal
Space group	R -3 :H
Unit cell dimensions	a = 22.4857(3) Å<br>b = 22.4857(3) Å<br>c = 11.0509(2) Å
Volume	4838.84(16) Å^3
Z	3
Density (calculated)	1.167 Mg/m^3
Absorption coefficient	1.695 mm^{-1}
F(000)	1701
Crystal size	0.20 × 0.20 × 0.10 mm^3
Theta range for data collection	3.932 to 73.162 °.
Index ranges	-26<=h<=27<br>-27<=k<=27<br>-13<=l<=12
Reflections collected	19985
Independent reflections	2152 (R_{int} = 0.0351)
Completeness to θ = 67.684 °	99.8 %
Absorption correction	Semi-empirical from equivalents
Max. and min. transmission	1.00000 and 0.89635
Refinement method	Full-matrix least-squares on F^2
Data / restraints / parameters	2152 / 241 / 205
Goodness-of-fit on F^2	1.121
Final *R* indices [I>2σ(I)]	$R1$ = 0.0584, $wR2$ = 0.1757
R indices (all data)	$R1$ = 0.0650, $wR2$ = 0.1845
Extinction coefficient	n/a
Largest diff. peak and hole	0.808 and -0.310 e.Å^{-3}

More details see CIF files.

Supplementary Table 2. Excess CO_2 uptake and active sites for **FJI-H14** and well-known MOFs

**Compounds**	**CO_2 uptake at 298 K and 1 atm** (cm^3 cm^{-3})	(cm^3 g^{-1})	**Concentration of active sites** mol L^{-1}	**Type of active sites**	**Ref**
**MAF-X27ox**	203	150	7.4	OMS, LBS	[1]
**MAF-X25ox**	196	160	6.9	OMS, LBS	[1]
**Co_2(dobdc)**	184[a]	156[a]	7.6	OMS	[2]
**FJI-H14**	171	146	9.2	OMS, LBS	This work
**Mg-MOF-74**	162	176	7.6	OMS	[3]
**UTSA-16**	160	96	5.3	H_2O	[4]
**SIFSIX-2-Cu-i**	151	121	2.2	SiF_6	[5]
**MPM-1-TIFSIX**	116	90	2.4	TiF_6	[6]
**Bio-MOF-11**	113	92	9.8	LBS	[7]
**Cu-TDPAT**	103	132	8.8	OMS, LBS	[8]
**Mg_2(dobpdc)**	103	144	3.5	OMS	[9]
**Mmen-CuBTTri**	100	94	3.1	LBS	[10]
**HKUST-1**	82	72	4.3	OMS	[11]
**PCN-88**	62[a]	94[a]	2.2	OMS	[12]

[a] 296 K.

Supplementary Table 3. MOFs with pore sizes larger than the kinetic diameter of N_2 shown restricted N_2 uptake at 77 K

**Compounds**	**Pore size** (Å)	**N_2 saturation uptake at 77 K** (cm^3 g^{-1})	**Ref**
**[Ni(bpe)$_2$(N(CN)$_2$)]$_n$**	6.9×4.8	0	[13]
**TOF-2**	13.7×14.7	0	[14]
**MPM-1-Cl**	6.2×6.2	18	[6]
**SMT-1**	7.4×10.7<br>6.7×12.5	near zero	[12]
**MFM-300(V^{III})**	6.7×6.7	no significant uptake	[15]

Supplementary Table 4. Heat of Adsorption Tabular Report for **FJI-H14**.

**CO_2 uptake (mmol g^{-1})**	**Slope**	**Y-Intercept**	**Adj. R-Square**	**Q_{st} (kJ mol^{-1})**	**Uncertainty (kJ mol^{-1})**
0.5	-3198.3	14.291±0.241	0.99715	26.592	0.580
1.0	-3249.4	15.118±0.253	0.99696	27.017	0.609
1.5	-3298.0	15.660±0.268	0.99668	27.421	0.645
2.0	-3340.5	16.074±0.279	0.99648	27.774	0.674
2.5	-3375.0	16.408±0.280	0.99654	28.061	0.675
3.0	-3415.2	16.732±0.285	0.99649	28.395	0.687
3.5	-3458.7	17.052±0.301	0.99618	28.757	0.727
4.0	-3502.9	17.368±0.311	0.99602	29.125	0.751
4.5	-3538.5	17.654±0.324	0.99577	29.421	0.782
5.0	-3584.5	17.983±0.339	0.99551	29.803	0.817
5.5	-3629.2	18.320±0.359	0.99507	30.175	0.866
5.9	-3665.4	18.605±0.385	0.99444	30.475	0.929

Supplementary Table 5. ESP charges calculated for **FJI-H14s**.

Atom	Cu1	O1	O2	O3	O4	N1
Charge (e)	0.798	-0.656	-0.614	-0.614	-0.656	0.409
Atom	N2	N3	N4	N5	N6	C1
Charge (e)	-0.492	-0.394	0.409	-0.394	-0.492	0.787
Atom	C2	C3	H3	C4	C5	C6
Charge (e)	-0.022	-0.135	0.119	-0.036	-0.022	-0.135
Atom	H6	C7	C8	C9	H9	C10
Charge (e)	0.119	-0.036	0.787	0.049	0.152	0.336
Atom	H10	C11	H11	C12	H12	
Charge (e)	0.098	0.049	0.152	0.336	0.098	

Note: The atom numbers see Supplementary Figure 14.

Supplementary Table 6. Bond length and ESP charge q for CO_2

$d_{C\text{-}O}$ (Å)	q_C (e)	q_O (e)
1.16	0.70	-0.35

Supplementary Table 7. Lennard-Jones parameters representing framework atoms and CO_2

	H	C	N	O	Cu	C_CO_2	O_CO_2
σ (Å)	2.846	3.473	3.263	3.033	3.113	2.80	3.05
ε/k_B (K)	7.649	47.856	38.949	48.158	2.516	27.0	79.0

Supplementary Reference

1 Liao, P.-Q. *et al.* Monodentate hydroxide as a super strong yet reversible active site for CO_2 capture from high-humidity flue gas. *Energy Environ. Sci.* **8**, 1011-1016 (2015).

2 Caskey, S. R., Wong-Foy, A. G. & Matzger, A. J. Dramatic Tuning of Carbon Dioxide Uptake via Metal Substitution in a Coordination Polymer with Cylindrical Pores. *J. Am. Chem. Soc.* **130**, 10870-10871 (2008).

3 Millward, A. R. & Yaghi, O. M. Metal−Organic Frameworks with Exceptionally High Capacity for Storage of Carbon Dioxide at Room Temperature. *J. Am. Chem. Soc.* **127**, 17998-17999 (2005).

4 Xiang, S. *et al.* Microporous metal-organic framework with potential for carbon dioxide capture at ambient conditions. *Nature Commun.* **3**, 954 (2012).

5 Nugent, P. *et al.* Porous materials with optimal adsorption thermodynamics and kinetics for CO_2 separation. *Nature* **495**, 80-84 (2013).

6 Nugent, P. S. *et al.* A Robust Molecular Porous Material with High CO_2 Uptake and Selectivity. *J. Am. Chem. Soc.* **135**, 10950-10953 (2013).

7 An, J., Geib, S. J. & Rosi, N. L. High and Selective CO_2 Uptake in a Cobalt Adeninate Metal−Organic Framework Exhibiting Pyrimidine- and Amino-Decorated Pores. *J. Am. Chem. Soc.* **132**, 38-39 (2010).

8 Li, B. *et al.* Enhanced Binding Affinity, Remarkable Selectivity, and High Capacity of CO_2 by Dual Functionalization of a rht-Type Metal–Organic Framework. *Angew. Chem. Int. Ed.* **51**, 1412-1415 (2012).

9 McDonald, T. M. *et al.* Capture of Carbon Dioxide from Air and Flue Gas in the Alkylamine-Appended Metal–Organic Framework mmen-Mg_2(dobpdc). *J. Am. Chem. Soc.* **134**, 7056-7065 (2012).

10 McDonald, T. M., D'Alessandro, D. M., Krishna, R. & Long, J. R. Enhanced carbon dioxide capture upon incorporation of N,N'-dimethylethylenediamine in the metal-organic framework CuBTTri. *Chem. Sci.* **2**, 2022-2028 (2011).

11 Min Wang, Q. *et al.* Metallo-organic molecular sieve for gas separation and

purification. *Microporous Mesoporous Mater.* **55**, 217-230 (2002).

12 Li, J.-R. *et al.* Porous materials with pre-designed single-molecule traps for CO_2 selective adsorption. *Nature Commun.* **4**, 1538 (2013).

13 Maji, T. K., Matsuda, R. & Kitagawa, S. A flexible interpenetrating coordination framework with a bimodal porous functionality. *Nature Mater.* **6**, 142-148 (2007).

14 Ok, K. M., Sung, J., Hu, G., Jacobs, R. M. J. & O'Hare, D. TOF-2: A Large 1D Channel Thorium Organic Framework. *J. Am. Chem. Soc.* **130**, 3762-3763 (2008).

15 Lu, Z. *et al.* Modulating supramolecular binding of carbon dioxide in a redox-active porous metal-organic framework. *Nature Commun.* **8**, 14212 (2017).
