# Supplementary material for: Carbon dioxide capture and conversion by an acid-base resistant metal-organic framework
Source: Nat Commun. 2017 Nov 1;8:1233. doi: 10.1038/s41467-017-01166-3 (PMC5663901; doi:10.1038/s41467-017-01166-3)
Supplement: Supplementary file 2 — Description of additional supplementary files [file 41467_2017_1166_MOESM2_ESM.pdf]

**Description of Additional Supplementary Files**

File Name: Supplementary Data 1
Description: Crystal data of FJI-H14

File Name: Supplementary Data 2
Description: Structure of FJI-H14 for GCMC simulation
